# 3D-QSAR CoMFA study of some Heteroarylpyrroles as Possible Anticandida Agents

**DOI:** 10.4103/0250-474X.41447

**Published:** 2008

**Authors:** P. C. Sharma, S. V. Sharma, Archana Sharma, B. Suresh

**Affiliations:** University Institute of Pharmaceutical Sciences, Kurukshetra University, Kurukshetra-136 119, India; 1 School of Chemical Sciences and Pharmacy, University of East Anglia, Norwich, NR47TJ, UK; 2 J. S. S. College of Pharmacy, Rocklands, Ootacamund-643 001, India

**Keywords:** CoMFA, 3D-QSAR, pyrroles, anticandida activity

## Abstract

A three dimensional quantitative structure-activity relationship study using the comparative molecular field analysis method was performed on a series of 3-aryl-4-[α-(1H-imidazol-1-yl) aryl methyl] pyrroles for their anticandida activity. This study was performed using 40 compounds, for which comparative molecular field analysis models were developed using a training set of 33 compounds. Database alignment of all 33 compounds was carried out by root-mean-square fit of atoms and field fit of the steric and electrostatic molecular fields. The resulting database was analyzed by partial least squares analysis with cross-validation; leave one out and no validation to extract optimum number of components. The analysis was then repeated with bootstrapping to generate the quantitative structure-activity relationship models. The predictive ability of comparative molecular field analysis model was evaluated by using a test set of 7 compounds. The 3D- quantitative structure-activity relationship model demonstrated a good fit, having r^2^ value of 0.964 and a cross validated coefficient r^2^ value as 0.598. Further comparison of the coefficient contour maps with the steric and electrostatic properties of the receptor has shown a high level of compatibility and good predictive capability.

Fungal infections in the human, range from superficial and cutaneous (such as dermatomycosis) to deeply invasive and disseminated (such as candidiasis and cryptococcosis) infections. In the past 20 years, fungal infections have increased dramatically-paradoxically, as risk advances. Fungal infections occur more frequently in people whose immune system is suppressed (because of organ transplantation, cancer chemotherapy, or the acquired immune deficiency syndrome) or who have been subject to invasive procedures (catheters, prosthetic device)^1^. Fungal infections are now important causes of morbidity and mortality among immuno-compromised hospitalized patients. The frequency of invasive candidiasis has increased ten-fold during the past decade^2^.

*Candida albicans* (CA) has been identified as the major opportunistic pathogen in the etiology of fungal infections; however, the frequency of other *Candida* species is increasing^3^. The current standard of therapies is the fungicidal (but toxic) polyene antibiotic, amphotericin B, and the safer (but fungistatic) azoles. In particular, the latter class of drugs is an important antifungal class widely used for AIDS-related mycotic pathologies^4^.

Quantitative structure activity relationship (QSAR) enables the investigators to establish a reliable quantitative structure-activity and structure-property relationships to derive *in silico* QSAR models to predict the activity of novel molecules prior to their synthesis. The overall process of QSAR model development can be divided into three stages namely, data preparation, data analysis, and model validation, representing a standard practice of any QSAR modeling. Successful application of 3D-QSAR methodologies have been used to generate models for various chemotherapeutic agents^5^,^6^.

We have carried out 3D-QSAR studies employing comparative molecular field analysis^5^ (CoMFA) techniques in order to study and gain further insight to deduce a correlation between structure and biological activity of 3-aryl-4-[α-(1H-imidazol-1-yl) aryl methyl] pyrroles as potent anticandida agents^7^.

In the CoMFA method, introduced by Crammer^8^,^9^, a relationship is established between the biological activities of a set of compounds and their steric and electrostatic properties. An advantage of CoMFA is its ability to predict the biological activity of molecules and represent the relationship between steric and electrostatic properties and biological activity in the form of contour maps^10^. An ‘active conformation’ of the ligands is generated and superimposed as per the predefined rules. These molecules are then placed in a box of predefined grid size. The steric and electrostatic interaction energy between each structure and a probe atom of defined size and charge are calculated at each grid point using the molecular mechanics force fields. A multivariate data analysis technique like partial least squares (PLS)^11^–^13^ is used to derive a linear equation from the resulting matrices. PLS is used in combination with cross validation to obtain the optimum number of components. This ensures that the QSAR models are selected on their ability to predict the data rather than to fit the data. The advantages of CoMFA studies are in the ability to predict the target properties of the compounds and to graphically present the QSAR in the form of coefficient contour maps^14^.

We present here 3D-QSAR studies using CoMFA method on a series of 3-aryl-4-[α-(1H-imidazol-1-yl) aryl methyl] pyrroles and the contour maps derived reveal the significance of steric and electrostatic fields. The structural variations in the molecular fields at particular regions in the space provide underlying structural requirements and 3D-QSAR models generated give good predictive ability and aid in the design of potent anticandida agents.

## MATERIALS AND METHODS

### Biological activity data:

**Fig. 1:**
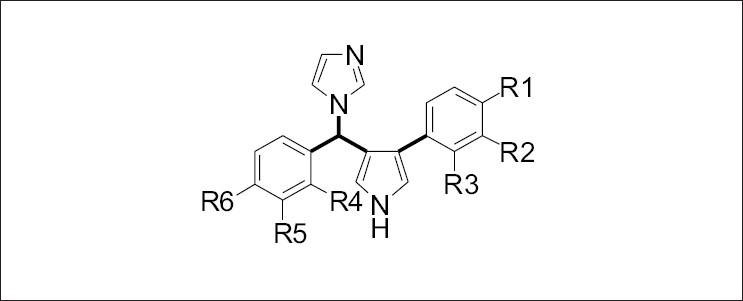
Heteroaryl pyrroles used for CoMFA study. General structure of compounds used for CoMFA study. Rotatable bonds selected for conformational analysis are shown in bold face

The antifungal activity data against *Candida albicans* for a series of 3-aryl-4-[α-(1H-imidazol-1-yl) aryl methyl] pyrroles containing 40 compounds as anticandida agents was used in this analysis. General structure of the compounds is shown in (fig. 1). Training set was formed by selecting 33 compounds from the original series. Test set compounds were no. 11, 12, 33, 34, 35, 37 and 42 (total 7 compounds), selected randomly. These compounds were not included in the analysis to generate the CoMFA model. The robustness and predictive ability of models were evaluated by selecting biological activity with chemical class similar to training set. CoMFA techniques were used to derive 3D-QSAR models for 3-aryl-4-[α-(1H-imidazol-1-yl)aryl methyl)pyrroles. The MIC data were used for the QSAR analysis as a dependent parameter, after converting to the reciprocal of the logarithm of MIC (pMIC) expressed in μM/ml (Table 1).

**TABLE 1:** EXPERIMENTAL ACTIVITIES OF MOLECULES USED IN TRAINING SET AND TEST SET

Compd No.	R^1^	R^2^	R^3^	R^4^	R^5^	R^6^	MIC μg/ml	MIC μM/ml	pMIC
1	H	H	H	H	H	H	53.9	0.180	0.744
2	H	H	H	H	H	Cl	47.3	0.142	0.847
3	H	H	H	H	H	F	71.2	0.224	0.648
4	H	H	H	H	H	CH_3_	133.3	0.425	0.370
5	H	H	H	H	H	NO_2_	56.7	0.164	0.782
6	H	H	H	H	H	NH_2_	142.4	0.453	0.343
7	H	H	Cl	H	H	H	43.8	0.130	0.885
8	H	H	Cl	H	H	Cl	18.7	0.050	1.292
9	H	H	Cl	H	H	F	48.3	0.137	0.861
10	H	H	Cl	H	H	CH_3_	35.4	0.102	0.991
11*	H	H	Cl	H	H	NO_2_	50.7	0.134	0.8722
12*	H	H	Cl	H	H	NH_2_	100.4	0.288	0.539
13	H	H	Cl	Cl	H	Cl	5.19	0.012	1.887
14	H	H	Cl	H	Cl	Cl	13.8	0.034	1.463
15	Cl	H	H	H	H	H	12.8	0.038	1.415
16	Cl	H	H	H	H	Cl	12.3	0.033	1.474
17	Cl	H	H	H	H	F	15.4	0.043	1.357
18	Cl	H	H	H	H	CH_3_	12.3	0.035	1.450
19	Cl	H	H	H	H	NO_2_	60.12	0.159	0.798
20	Cl	H	H	H	H	NH_2_	42.8	0.122	0.910
21	Cl	H	H	Cl	H	Cl	3.94	0.009	2.007
22	Cl	H	H	H	Cl	Cl	14.4	0.035	1.444
23	Cl	H	Cl	H	H	H	8.4	0.022	1.640
24	Cl	H	Cl	H	H	Cl	6.8	0.016	1.770
25	Cl	H	Cl	H	H	F	7.8	0.020	1.693
26	Cl	H	Cl	H	H	CH_3_	7.9	0.020	1.683
27	Cl	H	Cl	H	H	NO_2_	22.9	0.055	1.255
28	Cl	H	Cl	Cl	H	Cl	7.4	0.017	1.769
29	Cl	H	Cl	H	Cl	Cl	7.2	0.016	1.781
30	Cl	Cl	H	H	H	H	7.2	0.019	1.707
31	Cl	Cl	H	H	H	Cl	8.9	0.022	1.653
32	Cl	Cl	H	H	H	F	7.2	0.018	1.744
33*	Cl	Cl	H	H	H	CH_3_	7.9	0.020	1.683
34*	Cl	Cl	H	Cl	H	Cl	5.9	0.013	1.867
35*	Cl	Cl	H	H	Cl	Cl	2.2	0.021	1.677
36*	H	H	H	H	Cl	Cl	38.6	0.103	0.987
37	H	H	H	H	H	1pyrrole	30.4	0.081	1.091
38	H	H	Cl	H	H	1pyrrole	20.5	0.051	1.292
39	Cl	H	H	H	H	1pyrrole	119	0.29	0.537
40*	Cl	H	Cl	H	H	1pyrrole	33.7	0.078	1.107

* Compounds used in test set

### Molecular modeling:

A database of 33 compounds forming the training set was generated by molecular modeling. All molecular modeling and 3D-QSAR studies were performed using SYBYL 6.7^15^ with TRIPOS Force Field^16^ on a Silicon Graphics O_2_ workstation with IRIX operating system. The crystallographic data for these ligand complexes was not available hence all the molecules were constructed using a grid having a spacing of 1.54 A^°^ between grid points. This is the default spacing, which represents sp^3^ carbon-carbon bond length. The molecules were cleaned up and quick minimized after sketching. Because no experimental data on the biologically relevant conformations of the selected compounds were available (for example, atomic coordinates derived from X-ray crystallographic studies of their complexes with the putative receptor), we resorted to a general molecular mechanics approach (AM1) to build the conformational models to be used for generation of CoMFA models. A chirality check was performed to identify chiral atoms, after adding hydrogens, it was important to consider all possible enantiomers as the activity was reported for racemic mixtures. Then the molecules were subjected for energy minimization (geometry optimization) at a gradient of 1.0 kcal/mol with delta energy change of 0.001 kcal/mol with the TRIPOS standard force field. Structures were drawn by using default setting of SYBYL.

After conformational analysis (CA) by adopting AM1 Hamiltonian approach, the least energy conformation was selected, saved, and used for the charge calculation, assuming that it was the active one. We have used two different types of charges, calculated using the Gasteiger-Marsili method and the semi-empirical MOPAC method^17^.

### Partial Least Squares analysis (PLS):

The PLS analyses were done by following standard protocols^18^. In order to speed up the analysis and reduce the amount of noise, column filter was used by excluding the columns with a variance smaller than 2.0. Equal weights for the steric and electrostatic descriptors were assigned using the CoMFA scaling option.

### CoMFA Results:

Two CoMFA models were generated by using different types of partial atomic charges, results of which are shown in Table 2. Model A was derived using charges calculated according to Gasteiger-Marsili method, while Model B was obtained using MOPAC charges. From the results it can be observed that both the models are significant in term of their statistical acceptance, however model B was considered to be better due to higher correlation coefficient and Fischer’s statistical value.

**TABLE 2:** SUMMARY OF COMFA RESULTS

	Model A	Model B
R^2^_cv_	0.561	0.598
S E P	0.381	0.377
r^2^ conventional	0.886	0.964
S.E.	0.194	0.112
N_opt_	4	6
F value	58.344	126.195
*P* value	0.000	0.000
Steric contribution	0.418	0.428
Electrostatic contribution	0.582	0.572
r^2^_BS_	0.925±0.019	0.986±0.005
SD_BS_	0.051±0.017	0.046±0.007

Where r^2^CV is cross-validated r^2^, Nopt is optimum number of components, SEP is standard error of prediction, R^2^ convention is noncross-validated r^2^, SE is standard error of estimate, r^2^_BS_ is from 100 bootstrapping runs, F Value is Fischer static value, *P* Value is probability of r^2^=0 and SD_BS_ is standard deviation bootstrapping

### Prediction of Activity:

The 3-D QSAR analysis obtained as Model B was used for predicting the activity of the 33 compounds in the training set. The results are shown in Table 3. From the table, it can be seen that the predicted activities are very close to the experimental activities with minimum residual activity.

**TABLE 3:** PREDICTED AND EXPERIMENTAL ACTIVITIES OF TRAINING SET

Compd. No.	pMIC experimental	pMIC predicted	Residual activity*
1	0.74	0.72	0.02
2	0.84	0.77	0.07
3	0.64	0.65	0.07
4	0.37	0.38	-0.01
5	0.78	0.81	-0.03
6	0.34	0.32	0.02
7	0.88	1.03	0.15
8	1.29	1.22	0.06
9	0.86	0.90	-0.04
10	0.99	0.82	0.16
13	1.88	1.87	-0.01
14	1.46	1.59	-0.13
15	1.41	1.55	-0.13
16	1.47	1.38	0.08
17	1.35	1.32	0.03
18	1.45	1.25	0.19
19	0.79	0.87	-0.07
20	0.91	1.07	-0.16
21	2.00	1.97	0.03
22	1.44	1.37	0.07
23	1.64	1.70	-0.06
24	1.77	1.69	0.09
25	1.69	1.67	0.01
26	1.68	1.78	-0.10
27	1.25	1.28	-0.03
28	1.76	1.77	-0.01
29	1.78	1.77	0.01
30	1.70	1.68	0.02
31	1.65	1.69	-0.04
32	1.74	1.72	0.02
37	1.09	1.13	-0.04
38	1.29	1.19	0.01
39	0.53	0.64	-0.11

* Residual activity is the difference in predicted activity and experimental activity

### CoMFA contour maps:

The QSAR produced by CoMFA were represented as a 3-D coefficient contour map. To visualize the CoMFA steric and electrostatic fields from PLS analysis, contour maps of the product of the standard deviation associated with CoMFA column and coefficient (SD Coeff) at each lattice point were generated. The contour maps were plotted as percentage contribution to the QSAR equation and were associated with difference in biological activity. The CoMFA contour maps generated for model B were used to explain the structure activity relationship of antifungal drugs.

**Fig. 2:**
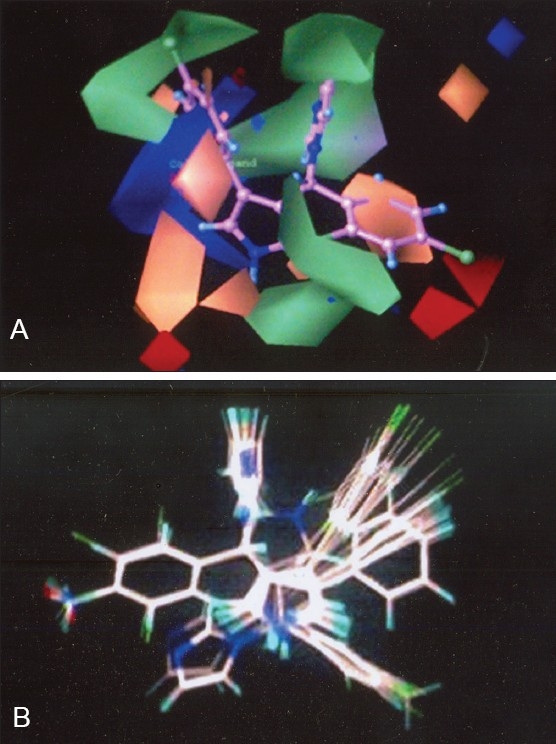
CoMFA steric and electrostatic contours field plot. Green contours in mesh view indicate regions where bulky groups increase activity, whereas yellow contours indicate regions where bulky groups decrease activity. Blue contours indicate where positive electrostatic groups increase activity, whereas red contours indicate where negative electrostatic groups increase activity. Compound 1 from training set is shown in fig. 2A and database alignment of 33 molecules of training set is shown in fig. 2B.

In CoMFA contour maps, the regions of high and low steric tolerance are shown in green and yellow polyhedra, respectively. CoMFA electrostatic field are shown as blue and red polyhedra in fig. 2. A low electron density within the inhibitors near blue and red polyhedra, respectively increase or decrease the activity.

## RESULTS AND DISCUSSION

The validity of Model B was further enhanced by bootstrapping process. Bootstrapping of 10 runs gave r^2^ of 0.986±0.005 with a very low standard error of 0.046±0.007 which added to the high confidence limit to this analysis. It can be seen that, both steric and electrostatic fields contributed to the QSAR equation by 42.8 and 57.2%, respectively. This suggested that variation in the antifungal activity is predominantly determined by electrostatic properties. Thus the results suggested that there is a good internal consistency in the data set generated in model B.

Model B performed exceptionally well in predicting the activity of most compound used in the test set. However, it must be emphasized that molecular alignment and conformations used in this study were selected in the absence of X-ray crystallographic coordinates of these molecules; still, the CoMFA model generated in the study showed very good prediction capability. From the Table 4, it can be observed that the predictions made using CoMFA model were satisfactory in most cases. In general, the percentage difference in the predicted activity of the synthesized compound ranges from 1.2 to 28.5%. The relative difference in the prediction is not unexpected and is within the acceptable limits.

**TABLE 4:** COMPARATIVE VARIATION IN THE EXPERIMENTAL AND PREDICTED ANTICANDIDA ACTIVITY OF COMPOUNDS IN TEST SET

Compd. No.	pMIC experimental	pMIC predicted	Residual activity	% Residual
11	0.87	0.89	-0.02	2.2
12	0.53	0.63	-0.10	15.8
33	1.68	1.69	-0.01	1.5
34	1.86	1.76	0.10	5.6
35	1.67	1.65	0.02	1.2
36	0.98	1.20	-0.22	18.3
40	1.10	1.54	-0.44	28.5

The data indicates the difference in predicted and experimental activities of compounds used in test set along with percentage residual activities. Predicted activities were obtained by using Model B

From these results, it is inferred that the 3-D QSAR model generated in this study has a potential to predict the activity of diverse compounds belonging to similar structural class. The investigations concerning the design of new chemical entities based on the proposed CoMFA models, predicting their antifungal activity prior to the synthesis would be part of our forthcoming communication.

## References

[1] Diamond RD (1991). The growing problem of mycoses in patients infected with the human immunodeficiency virus. Rev Infect Dis.

[2] Georgopapadakou NH, Walsh TJ (1994). Human mycoses: Drugs and targets for emerging pathogens. Science.

[3] Beck-Sague CM, Jarvis WR (1993). Secular trends in the epidemiology of nosocomial fungal infections in the United States, 1980-1990. J Inf Dis.

[4] Koltin Y (1990). Targets for antifungal drug discovery. Annu Rep Med Chem.

[5] Gokhale VM, Kulkarni VM (2000). Understanding the Antifungal Activity of Terbinafine Analogues Using Quantitative Structure-Activity Relationship (QSAR) Models. Bioorg Med Chem.

[6] Karki RG, Kulkarni VM (2001). Three-dimensional Quantitative Structure-Activity Relationship (3D-QSAR) of 3-Aryloxazolidin-2-one Antibacterials. Bioorg Med Chem.

[7] Artico M, Santo RD, Costi R, Massa S, Retico A, Artico M (1995). Antifungal Agents. 3-Ar l-4-[a-1(H -imidazol-l-yl)arylmethyl]pyrroles: A New Class of Potent Anticandida Agents. J Med Chem.

[8] Cramer RD, Patterson DE, Bunce JD (1988). Comparative molecular field analysis (CoMFA): Effect of shape on binding of steroids to carrier proteins. J Am Chem Soc.

[9] Clark M, Cramer RD, Jones DM, Patterson DE, Simeroth PE (1990). Comparative molecular field analysis (CoMFA) Toward its use with 3D structural databases. Tetrahedron Comput Met.

[10] Bhongade BA, Gadad AK (2004). 3D-QSAR CoMFA/CoMSIA studies on Urokinase plasminogen activator (uPA) inhibitors: A strategic design in novel anticancer agents. Bioorg Med Chem.

[11] Wold S, Ruhe A, Wold H, Dunn (1984). The collinearity problem in linear regression: The partial least square approach to generalized inverse. J Sci Stat Comput.

[12] Cramer RD, Bunce JD, Patterson DE (1988). Crossvalidation Bootstrapping and Partial Least Squares Compared with Multiple Regression in Conventional QSAR Studies. Quant Str Act Relat.

[13] Wold S, Albano C, Dunn WJ, Edlund U, Esbensen K, Geladi P, Kowalski BR (1984). Multivariate data analysis in chemistry. Chemometrics - Mathematics and Statistics in Chemistry.

[14] Puntambekar D, Giridhar R, Yadav MR (2006). 3D-QSAR studies of farnesyltransferase inhibitors: A comparative molecular field analysis approach. Bioorg Med Chem Lett.

[16] Matthew C, Cramer RD, Opdenbosch NV (1989). Validation of the general purpose tripos 5.2 force field. J Comput Chem.

[17] Gasteiger J, Marsili M (1980). Iterative partial equalization of orbital electronegativity-a rapid access to atomic charges. Tetrahedron.

[18] Geladi P (1988). Notes on the history and nature of partial least squares (PLS) modeling. J Chemom.

